# Exploring barriers to implementing adolescent pregnancy prevention interventions in low- and middle-income countries: Perspectives from international experts

**DOI:** 10.1371/journal.pone.0360943

**Published:** 2026-10-23

**Authors:** Aimable Nkurunziza, Jean Marie Vianney Nkurikiyintwali, Jean Pierre Ndayisenga, Yvonne Kasine, Innocent Twagirayezu, Yvonne Delphine Nsaba Uwera

**Affiliations:** 1 Faculty of Health Sciences, Arthur Labatt Family School of Nursing, Western University, London, Ontario, Canada; 2 Faculty of Education and Professional Studies, School of Nursing, Nipissing University, North Bay, Ontario, Canada; 3 Médecins Sans Frontières, West Africa and Central Africa, Abidjan, Ivory Coast; 4 School of Health Studies, Western University, London, Ontario, Canada; 5 Faculty of Nursing, University of Windsor, Windsor, Ontario, Canada; 6 School of Nursing and Midwifery, College of Medicine and Health Sciences, University of Rwanda, Kigali, Rwanda; University of West Florida, UNITED STATES OF AMERICA

## Abstract

**Background:**

Adolescent pregnancy remains a significant public health concern, particularly in low- and middle-income countries (LMICs). Although various interventions have been implemented to address this issue, many have failed to achieve positive outcomes due to numerous barriers.

**Objective:**

To explore the barriers to implementing adolescent pregnancy prevention interventions in LMICs.

**Methods:**

This qualitative exploratory study involved 19 adolescent pregnancy prevention program implementers across 24 LMICs. Data were collected through virtual in-depth individual interviews, organized using Dedoose software, and analyzed thematically.

**Results:**

Findings highlighted seven interrelated challenges shaping programme reach, acceptability, and sustainability. Experts reported that adolescents’ engagement depended on perceived usefulness and, at times, material or social benefits of attendance, while stigma made participation social risky and encouraged private information-seeking. Prevention efforts were further constrained by gendered allocation and unequal decision-making power, which limited boys’ participation and girls’ agency. Separately, poverty, household material insecurity, and transactional marriage practices such as bride price contributed to early unions and subsequent childbearing, while programme legitimacy is challenged by taboo, religious opposition, contraception myths, and the normalization of violence. Finally, implementation was hindered by administrative delays, weak institutional backing, school-level resistance, and unstable or poorly enforced policy environments, limiting continuity and scale-up of interventions.

**Conclusion:**

Effective teenage pregnancy prevention in LMICs requires more than delivering sexual and reproductive health (SRH) information; programmes must be designed to feel relevant to adolescents while reducing stigma and protecting confidentiality to support genuine engagement. Interventions also need to address gendered power dynamics and gatekeeper control, alongside community-level legitimacy barriers such as taboo, religious opposition, contraception myths, and normalized violence. Sustainable impact will depend on enabling implementation conditions, including reduced bureaucratic delays, stronger institutional and school support, coherent policies, reliable financing beyond donor cycles, and better data to guide targeted action.

## Introduction

Adolescents make up a significant portion of the global population, with most residing in low- and middle-income countries (LMICs) [1]. Adolescent pregnancy remains a major global health and development challenge, carrying far-reaching health, social, and economic consequences for adolescents and their communities, including adverse health outcomes, heightened vulnerability to unemployment, and disrupted educational trajectories (World Health Organization [2]. The WHO defines adolescent pregnancy as pregnancy occurring among girls aged 10–19 years [2,3]. While fertility rates among adolescents aged 15–19 have declined globally, progress has been uneven: adolescent birth rates in LMICs remain approximately four times higher than those in high-income regions, underscoring persistent and profound inequities [4,5].

As of 2019, it was estimated that each year, around 21 million pregnancies occurred among adolescents aged 15–19 in LMICs, with nearly half of these pregnancies being unintended and leading to approximately 12 million births [6]. Moreover, despite improvements in some settings, projections indicate that adolescent pregnancies may rise again by 2030, driven largely by the rapid growth of adolescent populations in many LMICs and the resulting demographic momentum. Trends in adolescent birth rates have varied across regions, with Southern Asia experiencing the steepest decline, while reductions have been slower in Latin America and the Caribbean (LAC) and sub-Saharan Africa (SSA) [7,8]. Although all regions have seen decreases, SSA and LAC still report the highest rates globally, at 97.9 and 51.4 births per 1,000 women, respectively, in 2023.

Global efforts have focused on improving the sexual and reproductive health rights (SRHR) of adolescents. However, progress has been uneven across countries, largely due to restrictive social norms that limit girls’ and women’s autonomy, gender stereotypes that discourage their participation in education and decision-making, and power imbalances that reduce their access to resources, opportunities, and health services in many LMICs. In these regions, inadequate infrastructure, widespread poverty, and high rates of gender-based violence have significantly hindered adolescents from accessing essential SRH services [1].

In response to these persistently high rates, numerous interventions have been implemented to prevent adolescent pregnancy across LMICs, including cash transfer programs, school-based education curricula, life-skills training, contraceptive provision initiatives, peer education programs, and mass media campaigns [9]. However, available evidence suggests that many initiatives do not consistently achieve their intended outcomes, even when they are designed to address key drivers of adolescent pregnancy. Importantly, while some studies have documented barriers encountered by those delivering interventions, these insights remain dispersed across contexts and intervention types, limiting the field’s ability to draw coherent, implementation-relevant lessons. There is therefore a critical need to systematically examine the barriers faced by key stakeholders involved in delivering adolescent pregnancy prevention interventions in LMICs. To our knowledge, no study has comprehensively examined this issue, making this research timely and significant. By synthesizing implementers’ perspectives, this study aims to strengthen understanding of real-world implementation constraints and to inform the design of more feasible, acceptable, and sustainable strategies for policymakers, practitioners, and researchers seeking to improve adolescent sexual and reproductive health outcomes in LMICs.

## Materials and methods

### Study design

This study employed a descriptive exploratory qualitative research design, which is particularly effective for gaining a comprehensive understanding of a specific area of interest [10]. This approach was appropriately selected to facilitate an in-depth exploration of the barriers experienced by stakeholders involved in implementing interventions aimed at addressing adolescent pregnancy in LMICs.

### Population and sampling

Purposive and snowball sampling was used to invite experts involved in the implementation of interventions aimed at preventing adolescent pregnancies across in LMICs. Eligible participants included individuals who had been involved in designing, implementing, or evaluating sexual and reproductive health interventions specifically targeting adolescent pregnancies in LMICs. Only those who had worked on such interventions for a minimum period of six months and were able to communicate in English and French were included.

The study initially aimed to recruit approximately 25 participants. Sample adequacy was assessed in relation to the study’s exploratory objective and the recurrence and depth of cross-cutting implementation concepts. Because participants represented diverse geographical, professional, and programme contexts, this assessment was made at the level of overarching programmatic and implementation barriers rather than within individual countries or regions. By the 19^th^ interview, the major categories were recurring across participants’ accounts, and the later interviews primarily elaborated existing categories or provided context-specific examples rather than introducing substantively new overarching categories. The dataset was therefore considered to have achieved conceptual or high-level thematic sufficiency for addressing the study objective. This determination does not imply that localized cultural meanings, intervention-specific experiences, or country- and region-level differences were exhaustively captured.

### Data collection instrument

An interview guide consisting of open-ended questions was developed by members of the research team based on literature [11–13]. Team members had extensive experience in qualitative research and reproductive health interventions. The interview guide included the following questions:

1. *Could you briefly describe your background and experience in implementing adolescent pregnancy prevention interventions in LMICs?*
2. *Based on your experience, what are the main barriers to implementing adolescent pregnancy prevention interventions in LMICs?*
3. *What strategies have been used to address or overcome these implementation barriers?*
4. *How have local context, culture, policies, and health system infrastructure influenced intervention implementation?*
5. *What are the key components of effective adolescent pregnancy prevention interventions in LMICs?*
6. *What recommendations would you offer to policymakers, practitioners, and program implementers to improve the implementation, scalability, and sustainability of these interventions?*
7. *Is there anything else you would like to add regarding barriers or facilitators to implementing adolescent pregnancy prevention interventions in LMICs?*

### Data collection procedures

Data collection was conducted between August and October 2024. Recruitment began with a targeted Google search designed to identify professionals with documented experience in the development, implementation, or evaluation of adolescent pregnancy prevention interventions in LMICs. The search combined topic-related terms, including “adolescent pregnancy prevention,” “teenage pregnancy prevention,” “adolescent sexual and reproductive health,” “youth sexual and reproductive health,” and “youth-friendly sexual and reproductive health services,” with role-related terms such as “programme implementer,” “programme manager,” “technical advisor,” “researcher,” “monitoring and evaluation specialist,” and “sexual and reproductive health advisor.” These terms were combined with geographical identifiers, including “LMIC,” “low- and middle-income countries,” regional terms such as “sub-Saharan Africa,” “Latin America,” and “Asia,” and the names of individual countries. Publicly available staff directories, project webpages, reports, and publication author affiliations were reviewed. Potential participants were screened to confirm that they had been involved in designing, implementing, or evaluating a relevant intervention for at least six months before they were contacted by email.

Since many researchers did not respond to our emails despite two reminders, we expanded our recruitment efforts by reaching out through platforms used by individuals who had implemented these interventions. Additionally, we connected with the study participants who helped link us with potential participants meeting the inclusion criteria. Those who agreed to participate were invited to take part in virtual semi-structured interviews. All interviews were conducted by the research team members via video conferencing (Zoom), and field notes were taken during and after each session. Data collection was concluded once saturation was reached. At the end of each interview, participants received a $20 CAD gift card as a token of appreciation to cover their time and internet expenses.

### Data analysis

All interviews were audio-recorded and transcribed verbatim by members of the research team. Interviews conducted in French were subsequently translated into English by bilingual members of the research team prior to analysis. Field notes were incorporated into the transcripts, which were then uploaded into Dedoose software to facilitate coding. Guided by thematic analysis framework [14–16], transcripts were thematically coded, and relationships between codes were identified. The research team members collaboratively reviewed the codes and held regular meetings to address and resolve any inconsistencies. Descriptive and explanatory accounts were developed to understand how context and setting influenced intervention implementation. Themes and categories were continuously compared and refined to ensure they accurately represented the data.

### Rigor

To ensure rigor, the study adhered to Lincoln and Guba’s four criteria [17]: credibility, dependability, confirmability, and transferability. Credibility was enhanced through prolonged engagement with participants, literature-informed interview guides, experienced data collectors, integration of field notes, conceptual sufficiency and regular team meetings. Dependability was maintained through an audit trail and team-based coding. The research team members conducted and transcribed the interviews, which enhanced the credibility and dependability of the findings by ensuring consistency in data collection and a deeper familiarity with the data. Confirmability was supported by reflexivity and routine debriefing sessions, while transferability was ensured through purposive sampling.

### Ethical considerations

Ethical approval for the study was obtained from the University of Rwanda, College of Medicine and Health Sciences Institutional Review Board (Approval Notice: No 508/CMHS IRB/2024). Individuals who expressed interest after receiving the invitation email were provided with a detailed information letter and given adequate time to review it. They were also encouraged to ask any questions prior to providing consent. Those who agreed to participate signed an informed consent form. Participants’ rights, including confidentiality and voluntary participation, were fully respected and protected throughout the study.

## Findings

### Socio-demographic data

Across the 19 study participants, the sample was nearly gender-balanced (10 female, 9 male) and represented a wide range of countries and settings, primarily across Africa, with additional participants from Latin America and Asia and one participant reporting work across multiple countries. Ages ranged from 27 to 60 years (mean ≈ 37.1 years), and professional experience ranged from 5 to 20 years (mean ≈ 11.1 years), indicating a generally mid- to senior-level workforce. Educational attainment was high overall, with 11 participants holding a master’s degree, 7 holding a bachelor’s degree, and 1 holding a PhD. Participants’ roles spanned clinical and leadership positions, including midwives, nurse-midwives, lecturers/university faculty, community health workers, program managers, executive directors, and SRH/youth health advisors (Table 1.).

**Table 1:** Socio-demographic data of the study participants (N = 19).

Country	Gender	Age (years)	Education	Field/Discipline	Role/Occupation	Experience (years)
Ghana	Female	34	Bachelor	Midwifery	Midwife	12
Brazil	Female	39	PhD	Midwifery	Assistant professor; Midwife	10
Bangladesh	Female	29	Bachelor	Midwifery	Midwife	5
Kenya	Male	36	Bachelor	Community health	Community health worker	11
Peru	Female	34	Master’s	Midwifery	Midwife	12
Uganda (1)	Female	36	Master’s	Midwifery	Clinical midwife; Lecturer	15
Gambia	Male	27	Bachelor	Social work	Social worker; Program manager	5
Tanzania	Female	36	Master’s	Nursing/Midwifery	Lecturer (nurse-midwife)	15
Niger	Male	37	Master’s	Medical	Gender & Youth Coordinator	10
Cameroon	Male	32	Master’s	Biomedical science	Executive director; Researcher	5
Uganda (2)	Male	36	Bachelor	Public health	Executive director; Parasocial worker	11
Nigeria	Female	41	Master’s	Social work	Executive director; Social worker	8
Eswatini	Male	60	Master’s	Public health	Program manager	15
Rwanda–Uganda	Female	44	Master’s	Midwifery	University lecturer	20
Namibia	Female	29	Bachelor	Public health	Army coordinator	5
Brazil, Venezuela, Democratic Republic of the Congo (DRC), Zimbabwe, Bangladesh, Afghanistan, Syria, Rwanda, and Central African Republic.	Male	32	Master’s	Midwifery	Adolescent & Youth Health Advisor	9
Ghana	Male	49	Master’s	Nursing	Registered nurse	20
Malawi (1)	Male	34	Bachelor	Public health	SRH Advisor	9
Malawi (2)	Female	39	Master’s	Social work	Social worker	13

### Themes

The thematic findings are organized into seven interconnected areas that reflect how expert implementers understand the realities of preventing teenage pregnancy in LMICs. First, experts highlighted that adolescents’ engagement is shaped by the extent to which interventions are perceived as useful and, in some instances, by what adolescents can gain from attending. Second, they described how stigma makes participation in SRH programmes socially risky, prompting some adolescents to avoid services and instead seek information privately, including through online sources. Third, experts emphasized that prevention efforts are constrained by gendered expectations and unequal decision-making power, which often limit girls’ agency and reduce boys’ participation. Fourth, they noted that limited autonomy and gatekeeping by parents, partners, and other authority figures restrict adolescents’ practical access to interventions. Fifth, implementers explained how economic pressure and entrenched social norms normalize early marriage and early childbearing, lowering the perceived need for prevention and reducing programme uptake. Sixth, they reported that interventions frequently face legitimacy challenges rooted in taboo, religious opposition, contraception myths, and the normalization of violence, all of which can generate community resistance. Finally, experts identified systemic implementation constraints, including administrative delays, weak institutional backing, school gatekeeping, and unstable policy environments that undermine the continuity, scale-up, and sustainability of pregnancy prevention programmes.

#### Theme 1: Engagement is not synonymous with uptake: Relevance and transactional participation.

Experts described a recurring mismatch between what programs are designed to achieve and why some adolescents show up. Several implementers observed that attendance does not always signal interest in pregnancy prevention or SRH learning; rather, participation can be driven by social and material benefits. One expert explained that adolescents could appear disengaged from the intended goals of the intervention but still attend because the program offered a space to connect with peers or access tangible support:

*However, they would still attend the interventions often not for the intended purpose, but to socialize, receive financial incentives, or access food. (P4).*

In contrast, other adolescents were perceived as not engaging because they did not see additional value in the intervention, especially when SRH topics had already been covered elsewhere. This “we’ve heard it before” perception was described as a barrier to sustained participation and deeper engagement:

*Some adolescents believe they have already received sufficient sexual health education in school and therefore do not see the value in engaging in further discussions on the topic. (P12).*

Together, these accounts highlight that implementers are not only trying to reach adolescents, but also trying to make prevention content feel timely, relevant, and worth the effort of participation.

#### Theme 2: Stigma makes SRH participation socially risky, pushing adolescents toward private information-seeking.

A strong thread across experts’ accounts was that adolescents often avoid SRH interventions not because they reject the information, but because participation can expose them to judgment. One implementer noted that adolescents may fear being labeled as sexually active simply for attending a program:

*Adolescents do not want to attend some programs/interventions because of stigma. They do not want anyone to know that they are getting SRH information. (P3)*

In response to this social risk, experts reported that some adolescents seek information in ways that feel more private and less visible. As one participant put it, adolescents may prefer searching online over engaging with trustable sources, even when the information quality is uncertain:

*You know, this generation is very different. Many adolescents prefer to seek information online or through social media rather than from parents, teachers, or healthcare providers.* P11

From an implementation perspective, these experiences suggest that stigma does not only reduce program participation; it can also shift adolescents toward channels where misinformation is more likely.

#### Theme 3: Gendered expectations and unequal power constrain prevention in ways intervention alone cannot solve.

Experts described pregnancy prevention as a gendered responsibility, with sexual and reproductive health programmes frequently perceived as being intended primarily for girls. This perception contributed to weaker engagement among boys and placed much of the responsibility for preventing pregnancy on female adolescents:

*Mostly, women or female students get involved. The male students are actually not willing to participate. (P6)*

At the same time, experts emphasized that that girls did not always have the authority to make decisions about programme participation or their sexual and reproductive health. Decision-making power was often held by male partners or other male authority figures:

For married adolescent girls, participation could also carry the risk of rejection or conflict within their relationships and communities::

*If a young girl is married off, it’s hard to convince her to come to our program because she fears being rejected by her husband or community. (P15)*

These accounts show that pregnancy prevention interventions operate within gendered relationships in which responsibility, authority, and social risk are unequally distributed. Boys may distance themselves from prevention programmes because they are framed as female spaces, while girls may be unable to act on prevention information because male partners or community expectations constrain their decisions.

#### Theme 4: Gatekeeping and limited autonomy restrict adolescents’ practical access to interventions.

Beyond beliefs and motivation, experts described practical restrictions on adolescents’ mobility and autonomy that prevent attendance and service use. In some settings, young people may require permission to leave home, turning participation into a gatekeeper-dependent activity rather than an individual choice. One implementer summarized this limitation bluntly:

*They don’t even have… rights to go outside themselves. So they need some consent for their parents to go outside.” (P5)*

Experts also highlighted that even when adolescents want to participate, families may block engagement due to distrust or misconceptions about program intent. One participant described broad family resistance:

*You want to help but families do not want or do not support their children to participate. (P2).*

Another explained that some families interpret SRH programming as encouraging sexual behavior rather than preventing harm:

*At times, the families would not allow the girl to participate in the program because they think that you are trying to introduce them to the sexual world… (P10).*

These experiences show how implementers often confront not only adolescent-level barriers, but also the authority structures surrounding adolescents that determine whether participation is allowed.

#### Theme 5: Economic insecurity and transactional marriage practices drive early unions and subsequent childbearing.

Experts described how poverty and material insecurity could make early marriage appear to families as an economic coping strategy rather than simply a culturally expected transition. In some settings, marriage arrangements were linked directly to anticipated financial or material benefits, including bride price:

*It’s their culture that she has to get married so that I can get the bride price.* (P1)

Participants also described situations in which caregivers arranged an adolescent girl’s marriage because they could not afford to meet household or material needs:

*They will give her [adolescent girl] out because they [parents] can’t afford to buy some stuff.* (P1)

Once girls entered early marriages, pregnancy was described as frequently occurring soon afterward:

*Families want to get their girls married off at an early age. So they get pregnant very soon.* (P2).

These accounts illustrate how early marriage and subsequent childbearing can become embedded in household survival strategies and economic exchanges. In such circumstances, pregnancy prevention programmes must compete with immediate material pressures that may make early marriage appear economically necessary or beneficial to families. Interventions focused only on knowledge or individual behaviour may therefore have limited influence unless they are accompanied by strategies that address household economic vulnerability and expand viable alternatives for adolescent girls.

#### Theme 6: Legitimacy challenges: Taboo, religion, contraception myths, and tolerated gender-based violence.

Experts described community contexts where SRH topics are socially restricted, making pregnancy prevention initiatives difficult to deliver openly. Some communities resist sexuality discussions with adolescents altogether:

*Many parents and elders in the community do not feel comfortable… discussing issues related to sexuality.* (P12).

Others view premarital sex discussions as inappropriate and offer no acceptable alternatives for prevention messaging:

*It is taboo to talk about premarital sex… and no alternatives are given.* (P15)

Religious opposition was described as another legitimacy barrier, with implementers reporting that SRH interventions may be rejected on doctrinal grounds:

*They say, ‘This is haram.’ It’s not according to what is supposed to be done.* (P16).

In addition, experts noted that credibility and belonging matter:

*You are not a Muslim, so you cannot interfere.* (P1)

These quotes show how implementers may be evaluated not only on what they deliver but also on who they are perceived to be.

Resistance also emerged around contraception specifically, where myths and fear undermine acceptance:

*They don’t buy into the idea of birth control because they think it will stop them from ever getting pregnant.* (P6)

Community discomfort with SRH education was described as strong enough that simply addressing sex education could provoke backlash:

*The community doesn’t believe in teaching young girls about sex.* (P10)

Experts also described environments where gender-based violence is normalized, compounding vulnerability and weakening prevention efforts. One participant framed gender inequality as an accepted oppressive influence expressed through violence:

*It’s an oppressive influence accepted in the community and is expressed by gender-based violence.* (P7)

Another account illustrated extreme tolerance of coercive marriage practices:

*Traditional rulers allowed girls to be kidnapped and married off…* (P1)

#### Theme 7: Delivery systems and sustainability: Administrative bottlenecks, weak institutional support, and unstable policy environments.

Experts described the work of implementing interventions as slowed by bureaucratic and governance constraints. One participant explained that approvals and certifications can involve repeated delays and sometimes corruption that consumes budgets before implementation begins:

*“If you land in the hands of those who are corrupt, they first want money… By the time you are reaching the right people, the money is already done or halfway.* (P16)

Another described the repeated effort required just to reach decision-makers:

*You need a lot of going and coming… Generally, bureaucratic processes slow down intervention programs.* (P8).

Even when permission is obtained, implementers reported limited tangible support from institutions expected to facilitate adolescent interventions. One expert described facilities as endorsing proposals but not providing practical resources:

*They have not provided financial or logistical support for adolescent interventions.* (P18)

Others emphasized that engaging healthcare workers may require additional training and incentives, which are not always available:

*Healthcare professionals require additional training and incentives to actively support adolescent reproductive health interventions.* (P8)

Schools, often critical for reaching adolescents, were described as difficult sites for implementation. Some institutions hesitate even when formally contacted:

*At times, we send letters to schools, and the authorities hesitate to accept.* (P6)

In other contexts, explicit restrictions limit what can be taught:

*[It] does not allow sex and sexuality to be explicitly taught in schools.* (P9)

Experts also pointed to policy and political barriers that undermine continuity and scale. Some described weak enforcement and follow-through:

*Policies exist on paper, but they are not always put into action.* (P12)

Others highlighted sustainability challenges linked to donor dependence and limited government budgeting:

*Our interventions depend on donor funding. If donor funding stops… the government does not allocate a budget to continue the programs.* (P6)

*[The project] stops when you leave due to the lack of the government’s will or funding to sustain it.* (P2).

Political opposition to contraception was also described as a direct threat to SRH programming:

*In our congress, we have a large group of people who strongly want to banish birth control.* (P4)

Finally, implementers described legal and coordination barriers that complicate service access and coherent implementation, including parental consent requirements. One participant noted,

*Laws do not allow adolescents to access healthcare without parental consent.* (P5).

Other participants noted conflicting sector directives as another barrier:

*One government, two ministries, and each has their own policies. The Ministry of Health says young people need SRH education from age 10, but the Ministry of Education does not support that.* (P15).

Another participant added,

*We have a clash between the Ministry of Education and the Ministry of Health, which is the agency responsible for rolling out health policies. The policy is such that students are not supposed to be introduced to sex and sexuality in secondary and junior secondary schools.* (P9).

Data limitations further constrained targeting and planning:

*We don’t have data disaggregated by age and sex, making it difficult to understand the prevalence of teenage pregnancy and appropriate interventions.* (P5).

Together, these accounts indicate that policy instability may result not only from absent or poorly enforced policies but also from contradictory directives across government sectors. Because the health and education sectors control different but interdependent components of adolescent SRH delivery, such contradictions can leave implementers without a clear and shared mandate and contribute to uneven school-level implementation.

## Discussion

The aim of this study was to explore the barriers encountered by stakeholders during the implementation of interventions aimed at preventing teenage pregnancy in LMICs. This study’s findings show that expert implementers view teenage pregnancy prevention in LMICs as constrained by a set of interconnected barriers that shape programme reach, acceptability, and sustainability.

Implementers described participation as conditional and sometimes transactional. Adolescents may attend sessions because of social interaction or material incentives, rather than because they perceive SRH content as useful. This may be related to the fact that accessing these services often involves costs, such as transportation fees or service charges, which may lead adolescents to attend only when material incentives are provided [18]. This distinction matters because attendance can be misconstrued as programme success when, in practice, adolescents may remain indifferent to the prevention objectives. Conversely, adolescents may disengage when messages are perceived as repetitive or disconnected from their lived realities (e.g., the sense that SRH has already been covered at school). However, studies in LMICs have found that adolescents often believe they already possess adequate SRH knowledge until they participate in these programs and engage meaningfully. Through this involvement, they ultimately enhance their SRH knowledge and skills [19,20]. These findings imply that prevention interventions must be designed not only to attract adolescents, but also to sustain meaningful engagement through content that is developmentally appropriate, context-specific, and responsive to adolescents’ actual concerns (e.g., relationships, safety, aspirations, social pressures), rather than relying primarily on information transfer or incentives. From an implementation standpoint, this also raises the need to clarify the role of incentives. While incentives may reduce participation barriers and promote initial contact, especially in resource-constrained settings, overreliance can unintentionally reinforce a “programme as benefit” dynamic that does not translate into internalized motivation or behavior change. Future intervention designs may therefore benefit from balancing practical supports (transport, refreshments, safe venues) with strategies that strengthen perceived relevance and skills acquisition, such as participatory approaches, peer-led components, and content that explicitly links pregnancy prevention to goals adolescents value (education continuity, future livelihoods, relationship wellbeing).

Experts described stigma as a central mechanism through which adolescents avoid SRH programmes and services. When participation signals sexual activity, or risks being interpreted that way, adolescents may protect their reputations by distancing themselves from formal SRH spaces. These findings align with those reported in a systematic review on access to and utilization of SRH services in African settings, which found that youth who experience stigma are less likely to use SRH services or may avoid seeking them altogether [18]. A notable implication is that stigma does not eliminate demand for SRH information; rather, it often redirects demand toward private channels (e.g., online searching), where privacy is higher but information quality may be uncertain [13]. This highlights a dual implementation challenge, including reducing the social costs of engagement and improving the credibility of the information adolescents access when they seek it privately. Interventions may need to strengthen confidentiality protections such as discreet entry points, trusted intermediaries, youth-friendly service hours, clear privacy messaging and shift from highly visible, stand-alone SRH programming toward integrated platforms that reduce identification (e.g., embedding SRH into broader youth wellbeing, life skills, or general health programming). In parallel, the preference for online information suggests a role for credible digital or hybrid approaches, such as moderated youth SRH platforms, verified social media content, or confidential helplines that meet adolescents where they already seek information while safeguarding accuracy. This is supported by a systematic review conducted in sub-Saharan Africa found that, despite challenges such as the digital divide, the cultural relevance of content, and the need for a thorough assessment of long-term impacts, delivering SRH information to adolescents through digital platforms is more effective than other program approaches [21].

Our findings suggest that effective adolescent pregnancy prevention interventions must go beyond the provision of information and services to actively address the gender norms that influence adolescents’ decisions and opportunities. These gendered norms not only influence participation in SRH initiatives but also contribute to unequal vulnerability. As girls are socialized into early marital and sexual roles, they face increased risk of exposure to HIV and other sexually transmitted infections, as well as heightened susceptibility to sexual coercion, exploitation, and violence [22]. Together, these dynamics illustrate how entrenched norms around femininity and masculinity not only restrict adolescents’ engagement with SRH services but also exacerbate the health and safety risks that disproportionately affect girls.

At the same time, decision-making power often rests with men (partners, fathers, male leaders), limiting girls’ capacity to act on SRH knowledge even when they possess it. However, a study conducted across 32 LMICs showed improvements in women’s decision-making regarding SRH. At the same time, the study revealed substantial disparities between countries, underscoring that women’s autonomy in SRH decisions is highly influenced by cultural and socioeconomic contexts [23]. These findings support the need to treat gender and power dynamics as core implementation considerations rather than peripheral context. Programmes may be strengthened by components that explicitly engage male adolescents, partners, and adult men; promote equitable norms; and create community-level accountability for protecting girls’ rights and wellbeing. Importantly, implementers’ experiences also indicate that interventions should anticipate resistance if they challenge gender hierarchies and should incorporate gradual, locally legitimate pathways for dialogue and norm change.

Experts described a consistent reality such as adolescents’ ability to attend programmes or access SRH services that is often contingent on permission from parents, guardians, partners, or other authority figures, consistent with findings from a systematic review in Sub-Saharan Africa [18]. Even when adolescents are motivated, they may lack autonomy to leave home, travel to services, or participate in programming, especially girls. This suggests that programme accessibility cannot be assessed solely by geographic availability; social permission and mobility constraints are equally decisive. Interventions may therefore need to incorporate family-facing and gatekeeper-facing engagement strategies, particularly in contexts where parental consent is legally required or socially expected. However, gatekeeper engagement must be approached carefully to avoid undermining adolescents’ confidentiality or creating additional surveillance [24,25]. A practical implication is the value of differentiated engagement, including building caregiver understanding and support for adolescent wellbeing (e.g., preventing school dropout, protecting health) while maintaining private, adolescent-controlled access to sensitive SRH services. Implementers may also benefit from flexible delivery models that reduce mobility barriers, such as school-based services where acceptable, mobile outreach with confidential pathways, or community-based safe spaces that are not explicitly labeled as SRH programmes.

Experts also highlighted how poverty, material deprivation, and transactional marriage practices can drive early unions and subsequent childbearing. Where households experience severe financial hardship, marriage may be viewed as a means of reducing caregiving costs, obtaining bride price, or responding to unmet material needs [23]. In such contexts, prevention messages that emphasize individual choice may have limited resonance because structural constraints shape what choices are feasible. This finding points to the importance of aligning pregnancy prevention efforts with broader social protection and opportunity-building strategies. While SRH education and services remain essential, they may be insufficient when families perceive early marriage as economically rational or culturally expected. Interventions may therefore be strengthened when linked to measures that reduce economic pressure (e.g., keeping girls in school, livelihood pathways, social support for vulnerable households) and when they address the social meaning of early marriage and fertility, not only the health risks [25].

Implementers reported that interventions often confront legitimacy barriers rooted in taboos around discussing sexuality, religious objections, misconceptions about contraception (e.g., fears of infertility), and normalization of gender-based violence (GBV) and coercive marriage. These barriers can create strong community pushback that shapes what can be delivered, where, and by whom [18]. The findings suggest that programmes may fail not because content is ineffective, but because delivery lacks local legitimacy or triggers moral and cultural resistance. This underscores the necessity of credibility-building as an implementation task. Strategies may include partnering with trusted local actors (including faith leaders where appropriate), using culturally resonant framing (e.g., protection, health, education continuation, family wellbeing), and proactively addressing contraception myths with clear, non-judgmental messaging. Where GBV and coercion are normalized, pregnancy prevention interventions also require safe referral pathways and protection-oriented partnerships, as SRH interventions alone may not address the underlying coercive conditions that drive risk. Additionally, implementers’ mention of language barriers signals that interventions must invest in accessible communication, local languages, appropriate translation, and culturally grounded materials to avoid exclusion and misunderstanding. These findings are consistent with, and further support, the results reported in previous studies [25–27].

Experts emphasized operational constraints that can erode programme continuity: bureaucratic delays, corruption, limited institutional or facility support beyond formal endorsement, challenges engaging health workers without training and incentives, school-level restrictions on SRH content, and unstable or poorly enforced policies. Many interventions were described as dependent on donor funding, with limited government budget lines to sustain programmes once external funding ends. These findings are consistent with the recent study in LMICs [25]. Implementers also noted gaps in age- and sex-disaggregated data that undermine planning, targeting, monitoring, and evaluation. These insights reinforce that pregnancy prevention interventions require more than good design; they require an enabling delivery ecosystem. Practical priorities include streamlining approvals, strengthening accountability and transparency, investing in workforce capacity for youth-friendly SRH delivery, and building stronger alignment between health and education sectors [25]. The sustainability concerns raised by implementers also point to the need for early planning for institutionalization: embedding programmes into existing service platforms, developing transition plans from donor to domestic financing, and strengthening routine data systems that allow decision-makers to identify where risks are highest and where interventions are working.

Finally, a particularly important systems-level finding was participants’ description of conflicting directives between the health and education sectors. While health authorities were reported to promote SRH education beginning at age 10, education-sector policies were described as restricting the explicit teaching of sex and sexuality in secondary schools. This represents a form of horizontal policy fragmentation, in which ministries responsible for interdependent components of adolescent pregnancy prevention operate under divergent mandates. Multisectoral governance literature in LMICs indicates that government sectors must coordinate around shared objectives to avoid conflicting policies and that successful collaboration depends on a common understanding of the problem, clearly assigned roles, aligned institutional incentives, distributed leadership, and appropriate financing and monitoring arrangements [28,29]. Addressing this systems-level bottleneck therefore requires more than a supportive policy within one ministry. Governments need formal inter-ministerial coordination structures, harmonized policy and operational guidance, clearly assigned responsibilities, joint training and implementation plans, shared financing and monitoring indicators, and mechanisms for resolving policy disagreements before programmes are introduced in schools.

### Strengths and limitations

A key strength of this study is its inclusion of experienced professionals involved in designing, implementing, or evaluating adolescent pregnancy prevention interventions across 24 LMICs. Participants represented diverse disciplines, professional roles, and geographic settings. Several limitations should also be considered. The use of purposive and snowball sampling, including recruitment through online searches, professional platforms, and participant referrals, may have introduced selection bias. Although the participants brought implementation experience spanning 24 LMICs, the sample was not designed to achieve saturation within individual countries or regions. The recurrence of broad programmatic and implementation barriers provided useful cross-contextual insights; however, localized cultural meanings, language-related considerations, health-system arrangements, policy environments, and intervention-specific processes may not have been fully captured. In addition, the sample was drawn primarily from African settings, with fewer perspectives from Latin America and Asia, which may limit the transferability of the findings across the full diversity of LMIC contexts. Restricting participation to individuals who could communicate in English or French may also have excluded relevant perspectives. Finally, the findings reflect implementers’ perspectives rather than the direct experiences of adolescents, families, or community gatekeepers, and the broad cross-country focus may not fully capture country-specific cultural, institutional, and policy differences.

Future research could build on these findings through region-specific analyses to further explore contextual differences in implementation experiences across LMICs. Studies should also incorporate the perspectives of adolescents, families, community and religious leaders, educators, healthcare providers, and policymakers to identify locally acceptable strategies for addressing stigma, gendered power relations, gatekeeping, economic pressures, and institutional barriers. Longitudinal and implementation-focused research is also needed to evaluate which context-sensitive approaches most effectively strengthen programme reach, acceptability, sustainability, and scale-up over time.

## Conclusion

Effective adolescent pregnancy prevention in LMICs requires more than the provision SRH information. Interventions should be informed by thorough context and needs assessments to identify potential barriers and guide the development of appropriate implementation strategies. Programmes must be designed to resonate with adolescents, reduce stigma, safeguard confidentiality, address gender and power-related barriers, and tackle misconceptions surrounding contraception and adolescent sexuality. Strengthening advocacy, community engagement, and stakeholder support is critical for improving programme acceptability and sustainability. Long-term success will also depend on supportive policies, adequate financing, efficient implementation processes, and robust data systems to inform evidence-based decision-making and programme improvement.
